# Conditional loss of ERK1 and ERK2 results in abnormal placentation and delayed parturition in the mouse

**DOI:** 10.1038/s41598-019-45997-0

**Published:** 2019-07-03

**Authors:** Jessica L. Brown, Jennifer L. Sones, Cynthia N. Angulo, Keelin Abbott, Andrew D. Miller, Ulrich Boehm, Mark S. Roberson

**Affiliations:** 1 Department Biomedical Science, College of Veterinary Medicine, Cornell University, Ithaca, NY USA; 2 Department Veterinary Clinical Sciences, School of Veterinary Medicine, Louisiana State University, Baton Rouge, LA USA; 3 Experimental Pharmacology, Center for Molecular Signaling (PZMS), Saarland University School of Medicine, Homburg, Germany

**Keywords:** Reproductive biology, Extracellular signalling molecules, Development

## Abstract

Extracellular-signal-regulated kinases (ERK) 1 and 2 regulate many aspects of the hypothalamic-pituitary-gonadal axis. We sought to understand the role of ERK1/2 signaling in cells expressing a Cre allele regulated by the endogenous *GnRHR* promoter (GRIC-ERKdko). Adult female GRIC-ERKdko mice were hypogonadotropic and anovulatory. Gonadotropin administration and mating led to pregnancy in one-third of the ERKdko females. Litters from ERKdko females and pup weights were reduced coincident with delayed parturition and 100% neonatal mortality. Based on this, we examined Cre expression in implantation sites as a potential mechanism. *GnRHR* mRNA levels at e10.5 and e12.5 were comparable to pituitary levels from adult female mice at proestrus and *GnRHR* mRNA in decidua was enriched compared to whole implantation site. *In vivo* studies confirmed recombination in decidua, and GRIC-ERKdko placentas showed reduced ERK2 expression. Histopathology revealed abnormalities in placental architecture in the GRIC-ERKdko animals. Regions of apoptosis at the decidual/uterine interface at e18.5 were observed in control animals but apoptotic tone in these regions was reduced in ERKdko animals. These studies support a potential model of ERK-dependent signaling within the implantation site leading to loss of placental architecture and mis-regulation of apoptotic events at parturition occurring coincident with prolonged gestation and neonatal mortality.

## Introduction

Mitogen-activated protein kinases (MAPKs) are signal transducing kinases that contribute to cell differentiation, survival and proliferation^1,2^. MAPKs are a part of ubiquitous signaling cascades involving multiple intermediates associated with phospho-transfer including Raf-1 kinase, MEK kinase (MEKK)1, MAPK-ERK kinases (MEK) 1 and 2, and extracellular signal regulated kinases (ERK) 1 and 2 (also referred to as MAPK3 and MAPK1, respectively)^3,4^. ERK1 and ERK2 have been long known to function integrally during embryonic development and in adult animals in a number of physiologically and pathophysiologically relevant conditions^5,6^. During development, ERK1 and ERK2 have divergent functions since ERK1 null mice are grossly viable and fertile while ERK2 null mice are embryonic lethal^7–9^. Embryonic lethality of the ERK2 null mice appears to be strain-specific and/or related to the design of the knockout allele; some having reported evidence of failed early mesodermal differentiation while others reporting mis-regulation of placental development^10,11^. Both instances of ERK2 loss lead to early embryo mortality where ERK1 does not appear to be compensatory. In adult animals, ERK1 and 2 appear largely redundant in coordinating cellular responses to growth factors, peptide hormones and other ligands via a wide array of receptor subclasses controlling proliferation, differentiation and survival depending on physiological and cellular context.

Since ERK2 null animals are embryonic lethal, a conditional approach to examining the specific role of ERK signaling in discrete cell types *in vivo* is necessary. In past studies, we reported a pituitary-specific conditional ERK double knockout (ERKdko) using the ERK1 null and ERK2 floxed alleles combined with a Cre recombinase driver regulated by the gene promoter for the α subunit to the glycoprotein hormones (αGSU)^3,4^ and the gonadotrope-specific Cre driver (GRIC)^12,13^. These studies were instrumental in understanding the role and requirement for GnRH-induced ERK signaling to a cohort of immediate early response genes regulating gonadotropin biosynthesis in pituitary gonadotropes. The resultant phenotype was female-specific anovulatory infertility due to a loss of luteinizing hormone (LH) β subunit expression resulting in an inability to mount a preovulatory surge of LH. Other gonadotrope-specific genes (αGSU, follicle stimulating hormone (FSH) β and the GnRHR) are also impacted when ERKs are specifically deleted in this cell type^3,12^. Further, we examined the specific role of Raf-1 kinase in the regulation of GnRH-induced ERK activation finding that Raf-1 was dispensable for activation of this pathway. Similar studies of ERK double knockout conditionally in GnRH neurons did not impact fertility^14^ while loss of ERKs in the granulosa cell compartment revealed a requirement for ERK signaling in the ovarian follicle and loss of LH-induced ERK activation in granulosa cells resulted in infertility^15^. Thus, ERK signaling across the reproductive axis plays a key role in the endocrine–mediated control of fertility in female mice.

Given that in the GRIC model, Cre recombinase is expressed under the control of the endogenous *GnRHR* promoter, we also need to consider expression of GnRHR at other possible sites that could influence the overall impact of ERKdko using this Cre driver. GRIC expression has been identified in the male germ cell lineage complicating interpretation of the discrete role of ERK loss in the pituitary versus the testis. We recently described the role of ERK loss within the gonadotrope and the spermatogonial lineage, effectively parsing out the impact of ERK loss at both levels of the reproductive axis during aging^12^. Based upon this observation of non-gonadotrope Cre expression, we were particularly vigilant in assessing GRIC-mediated gene recombination in other tissues, such as the placenta.

In the present study, we again make use of conditional deletion of ERK1 and 2 using the GRIC driver to examine the hypothesis that these hypogonadotropic-hypogonadal animals are capable of responding to exogenous gonadotropins, ovulate and establish pregnancy. A compelling part of this experimental question was whether the ERKdko females could support *corpora lutea* function following induced ovulation and during early pregnancy given the limited biosynthesis and secretion of LH in the GRIC ERKdko mouse model. We show that female mice harboring ERK gene deletions directed by the GRIC Cre driver are capable of establishing and carrying pregnancy to term. Further, Cre-mediated recombination was observed within the maternal decidua and placenta and while presently speculative, loss of ERKs within these compartments may be associated with marked abnormalities within the implantation site and a prolonged gestational phenotype and complete neonatal lethality.

## Results

### ERKdko animals can ovulate in response to exogenous gonadotropin stimulation

**Figure 1:**
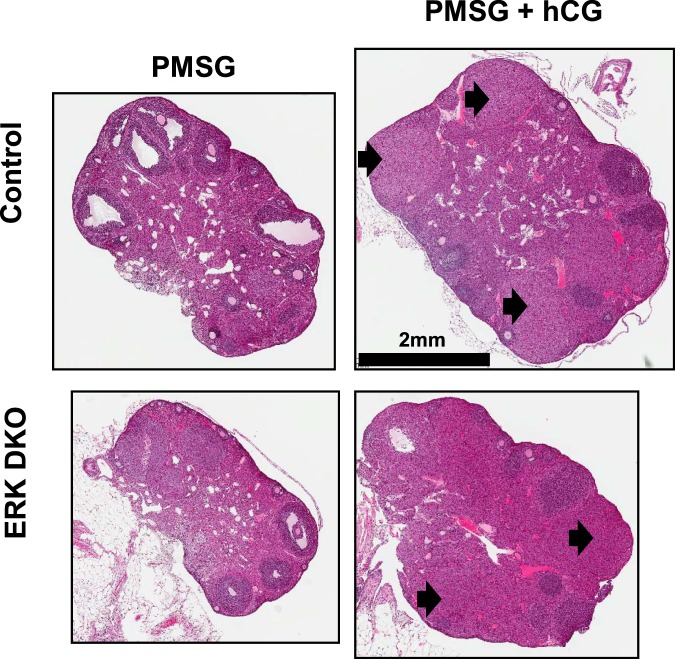
ERKdko females respond to exogenous gonadotropin stimulation leading to ovulation. Photomicrographs of ovaries from Control and ERKdko female mice following administration of PMSG or PMSG and hCG. Magnification bar is shown and arrows identify some of the *corpora lutea*.

We mated the ERK2^fl/fl^, ERK1^−/−^ mice with GRIC animals to improve specificity of ERK ablation to the pituitary gonadotropes compared with previous use of an αGSU-Cre driver^3,13^. In the present studies, these animals were designated ERKdko animals while control animals were ERK2^fl/fl^, ERK1^−/−^, but Cre negative. Consistent with the αGSU model system^3^, female ERKdko GRIC mice were infertile and anovulatory, with blunted gonadotropin secretion in response to GnRH following ovariectomy consistent with previous studies (not shown). To better understand the gonadal response to exogenous gonadotropin stimulation in this model, female control and ERKdko animals were treated with either pregnant mare serum gonadotropin (PMSG) or a standard superovulation protocol of PMSG followed by administration of human chorionic gonadotropin (hCG) to induce ovulation^16^. Three days later, control and ERKdko females were euthanized and ovaries were collected and the presence of *corpora lutea* (CL) determined histologically. As expected, addition of hCG in the superovulation paradigm resulted in the presence of CLs in both control and ERKdko females compared with PMSG alone (Fig. 1). These data support the conclusion that the ERKdko females are fully capable of ovulation when provided exogenous gonadotropin stimulation.

### ERKdko animals can maintain pregnancy, but show prolonged gestation and parturition

We next investigated whether superovulated ERKdko females could establish and maintain a pregnancy to term with viable pups. Control and ERKdko females were treated with PMSG/hCG, paired with control fertile males and were observed for copulatory plugs. In this experimental paradigm, all control and ~70% of ERKdko animals exhibited copulatory plugs. Control and ERKdko females with a copulatory plug were allowed to proceed through gestation without any human interference until near expected term. Beginning in the morning of e18.5, females were observed visually every 12 hours and assessed for behavioral signs of parturition (hiding, contractions, and visible distress) or the presence of pups. Control animals gave birth normally on gestational day 20.3; however, ERKdko females did not give birth until gestational day 23.9 (p < 0.05; Table 1). In control females, parturition was complete within a 12 hour period; while ERKdko females showed continuous behavioral signs of labor for 2.3 days (p < 0.05; Table 1) suggesting the possibility of abnormal parturition or dystocia. Finally, control animals had an average of 9 pups/litter; while litter size in the ERKdko females was 2.4 pups/litter (p < 0.05; Table 1). Pups from ERKdko females were stillborn or died soon after birth; on postnatal day 3, the percentage of live pups from the ERKdko females was zero compared with 85.7% in the control females (p < 0.05; Table 1).

**Table 1:** Reproductive characteristics of control and GRIC ERKdko female mice.

	Litter size (# of pups)	% live pups on PN3	Gestation length (d)	Parturition length (d)
Control	9.0 ± 1.0^a^ (n = 3)	85.7 ± 11.7^a^ (n = 3)	20.3 ± 0.25^a^ (n = 5)	1.0 ± 0.0^a^ (n = 4)
ERK DKO	2.4 ± 1.3^b^ (n = 7)	0^b^ (n = 7)	23.9 ± 0.55^b^ (n = 7)	2.3 ± 0.42^b^ (n = 4)

Data are presented as mean ± standard error of the mean and sample size shown in parentheses. Differing superscripts (a, b) within columns are different (p < 0.05).

### GnRHR is preferentially expressed in the placental decidua in early gestation

The literature suggests healthy viable pups are routinely born to wildtype females after superovulation protocols suggesting administration of gonadotropins to induce ovulation was not likely a cause of prolonged gestation and neonatal demise in our model system^16–18^. Moreover, while ERKdko animals have been shown to display defects in gonadotropin production, no evidence in the literature directly links hypogonadotropism to delayed parturition and periparturient mortality^19–22^. We considered that the abnormal pregnancies observed may be a consequence of Cre-mediated recombination and subsequent loss of ERK signaling at the maternal/fetal interface, as GnRH and GnRHR have been detected in the placenta and decidua of several species^23–28^.

**Figure 2:**
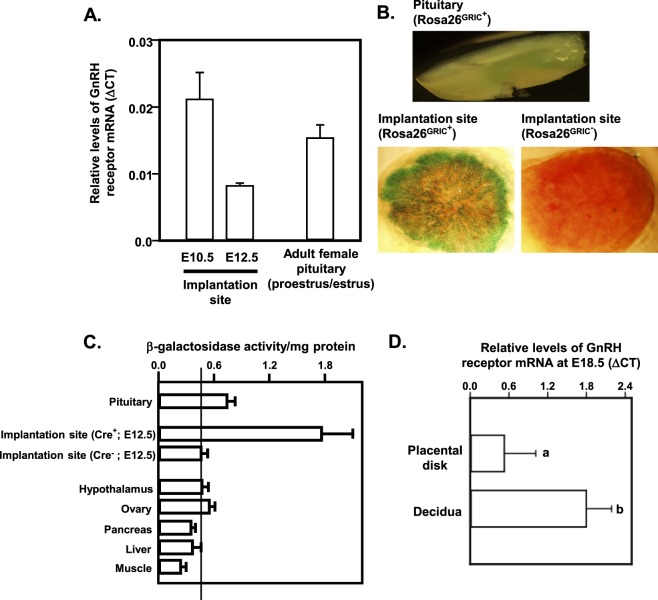
GnRH receptor (R) mRNA is detectable in the placental disk at embryonic (e) days 10.5 and 12.5 and the GRIC Cre mediates recombination in maternal decidua and placenta. (**A**) RT-PCR was performed on placental disks from embryonic days 10.5 and 12.5. Levels of GnRHR were comparable with those seen in adult female pituitaries collected at proestrus/estrus. N = 3 placentas/gestational age. In (**B**) Adult ROSA26^GRIC+^ mice expressing a β-galactosidase (β-gal)-GFP fusion protein show β-gal activity in the female pituitary *in situ*. β-gal staining indicates the presence of GRIC Cre activity in placenta from e18.5 ROSA26^GRIC+^ embryos but not placentas from e18.5 ROSA26^GRIC−^ embryos. (**C**) Liquid β-gal activity assay confirmed GRIC Cre-mediated recombination in ROSA26^GRIC+^ pituitaries and placentas. ROSA26^GRIC−^ placental disk and ROSA26^GRIC+^ hypothalamus, ovary, pancreas, liver and muscle were used as controls. (**D**) qRT-PCR showed enrichment of GnRHR mRNA expression in placental decidua compared to whole placental disk. Differing letters (a and b) over individual bars identify differences (p < 0.05). N = >4 placentas/genotype.

We used qRT-PCR to detect GnRHR mRNA in implantation sites at e10.5 and e12.5 mice and compared this directly with female pituitaries at proestrus/estrus (Fig. 2A). Levels of GnRHR mRNA in these tissues were consistent with levels of GnRHR found in adult female pituitaries at proestrus/estrus. To further validate these observations, we used the Rosa26 reporter mouse line mated to GRIC^+^ males. In this model, Cre-mediated recombination results in expression of a β-galactosidase (β-gal)-green fluorescent protein (GFP) fusion protein. *In situ* β-gal staining of whole mouse pituitaries and implantation sites confirmed recombination and β-gal activity within both tissues in ROSA^GRIC+^ mice. The highest density of β-gal activity was found at the periphery of the implantation site, indicating potential enrichment within the maternal decidua (Fig. 2B). Using placental tissues from ROSA26^GRIC−^ and ROSA26^GRIC+^ animals obtained at e12.5, we then carried out an *in vitro* β-galactosidase activity assay to quantify relative β-gal expression in a cross section of tissues (Fig. 2C). We compared ROSA26^GRIC−^ and ROSA26^GRIC+^ placental tissues along with ROSA26^GRIC+^ pituitary, hypothalamus, ovary, pancreas, liver and muscle from the same animals. Placenta lysates from ROSA26^GRIC+^ animals revealed a fourfold increase (p < 0.05) of β-gal activity compared with the ROSA26^GRIC−^. To further parse out localization of GnRHR expression within the implantation site, e12.5 placentas were dissected to carefully separate and enrich maternal decidua from the remainder of the placental disk including the chorionic plate and labyrinth. RNA was extracted and qRT-PCR was performed, allowing us to quantify enrichment of the GnRHR mRNA in the decidua versus the remainder of the disk at e12.5. GnRHR mRNA was more abundantly expressed in the maternal decidua compared to the remainder of the placental disk where GnRHR mRNA was lower but clearly detectable (Fig. 2D).

### GnRHR is expressed primarily in decidua at e12.5 and in the labyrinth at e18.5

Rosa26 animals were time mated with GRIC^+^ males, and euthanized at e12.5 and e18.5. Placentas were collected, snap frozen, serial sectioned and stained for GFP expression. At e12.5, GFP expression appeared to be expressed most abundantly in the decidua (Supplemental Fig. 1A,B). Punctate perinuclear GFP immunoreactivity was detected in clusters of cell within the decidua. There is modest basal expression of GFP throughout the cytoplasm of the labyrinth, but no areas of punctate expression were observed. At e18.5, punctate expression in the decidua was no longer observed; however, the labyrinth showed increased expression of GFP when compared to e12.5. Importantly, GFP was not detected within the junctional zone at e18.5 demonstrating the specificity of GFP expression within decidua and labyrinth. Negative controls (secondary antibody alone) for the immunofluorescence define the detection limit of the assay.

### Cre-mediated recombination results in loss of placental ERK2

Western blot analyses added additional confirmation of Cre-mediated *ERK* deletion within the decidua/placental disk at E12.5 (Supplemental Fig. 2A,B). We specifically assayed for the abundance of ERK2 since our genetic model is null for the ERK1 allele. For the remainder of the characterization, we separated the animals into three categories: control/control (control dam, control pup), ERKdko/control (ERKdko dam, control pup), and ERKdko/ERKdko (ERKdko dam, ERKdko pup). We observed ~30% reduction of ERK2 protein levels in decidua/placentas from both ERKdko groups when compared to placentas from control dams at e12.5. This suggests that the genotype of the dam may have a more significant impact on ERK expression than the genotype of the pup, again consistent with recombination ERK2 in decidua or other cells types within the uterus at this gestational age.

### Loss of ERK2 in the implantation site occurred coincident with fetal growth defects and gross and histological abnormalities in the placental disk

**Figure 3:**
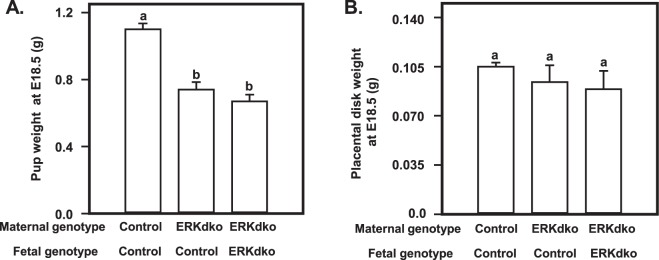
Pups from GRIC ERKdko females have reduced fetal weight at e18.5. Fetal (**A**) and placental (**B**) weights are shown at e18.5 from control/control, GRIC ERKdko/control and GRIC ERKdko/GRIC ERKdko pups and placentas, respectively. N = >4 placentas/genotype. Differing letters (a and b) over individual bars identify differences (p < 0.05).

To examine the impact of ERK loss at the maternal fetal interface, ERKdko and control females were superovulated, mated, euthanized at e18.5 and pups and placental tissues dissected. Anecdotally, all pups from the ERKdko and control dams were noted to be alive at this gestational age and euthanized humanely. ERKdko/control (maternal/fetal) and ERKdko/ERKdko pups displayed reduced fetal weight at e18.5 compared to control/control pups (p < 0.05; Fig. 3A). Consistent with earlier studies on ERK2 expression levels, reduced pup weight was primarily associated with loss of ERKs within the decidua (maternal ERKdko) at this time point. Intrauterine growth restriction seen in ERKdko/control or ERKdko/ERKdko pups could not be accounted for by changes in placental weight (Fig. 3B) indicating the possibility that fetal growth restriction observed was due to loss of relative placental efficiency (mass of fetus/mass of placental disk), not strictly placental size.

**Figure 4:**
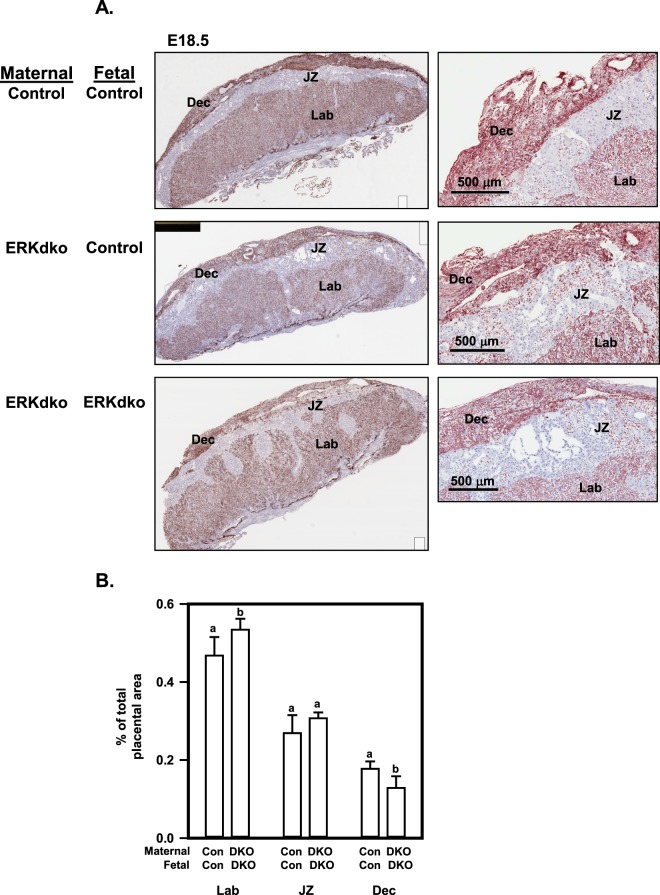
Histological assessment of placental disks in the GRIC ERKdko model reveals abnormal architecture. (**A**) Representative sections from control/control, GRIC ERKdko/control and GRIC ERKdko/GRIC ERKdko placentas at e18.5 were stained for isolectin B4 to mark the glycocalyx associated with blood vessel. Panels on the left are low magnification while panels on the right are higher magnification. The decidua (Dec), junctional zone (JZ) and labyrinth (Lab) are indicated. The magnification bar is shown for the higher magnification sections on the right. (**B**) Quantitation of decidual, junctional zone and labyrinth are reported as % of total placental disk area in control (Con) and GRIC ERKdko (DKO) animals. N = 3 placentas/genotype. Differing letters (a and b) over individual bars identify differences (p < 0.05).

Histological assessment of these implantation sites at e18.5 is shown in Fig. 4. For these studies, placentas were dissected free from the uterine wall, fixed in formalin, then sectioned and stained with isolectin B4 to identify the glycocalyx associated with blood vessels and lightly counter stained with hematoxylin/eosin. This approach clearly demarcates labyrinth, junctional and decidual compartments within the disk. Pups from ERKdko dams had abnormal placental architecture regardless of fetal genotype at e18.5 (Fig. 4A). Figure 4B depicts the distribution of the area of the labyrinth, junctional zone and decidua which varied with genotype. Using Aperio image analysis software, control/control (maternal/fetal) placentas were characterized by approximately 49% labyrinth, 26% junctional zone, and 17% decidua (with the remainder of the area attributed to chorionic plate). ERKdko/ERKdko placentas showed a modestly enlarged labyrinth and a smaller decidual area (Fig. 4B). ERKdko/control placentas showed an intermediate phenotype that was not significantly different from either of the other two genotypes. Pathological findings indicated that placentas from ERKdko dams and control fetuses appeared histologically disorganized compared to control placentas, displaying variability in decidual thickness. There were fingerlike projections of the junctional zone protruding into the labyrinth. GRIC-mediated recombination of ERK2 was associated with the appearance of large acellular spaces within the junctional zone. These findings were further exacerbated in the ERKdko/ERKdko placentas, with more marked histological changes, especially in the decidua and junctional zone, consistent with greater disorganization of the normal placental architecture seen in the control/control placentas.

### Histological changes in the placenta occur coincident with altered placental isolectin B4 binding

**Figure 5:**
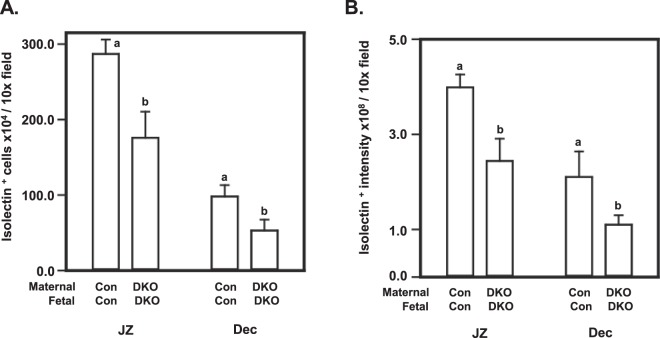
Assessment of isolectin B4 binding in GRIC ERKdko mice implantation sites. The number of isolectin B4^+^ cells (**A**) and staining intensity (**B**) are reported for control/control and GRIC ERKdko dam/GRIC ERKdko groups at e18.5 as number of cells or relative staining intensity (pixel intensity)/10 x magnification field. N = >4 placentas/genotype. Differing letters (a and b) over individual bars identify differences (p < 0.05).

Since endothelial cell dysfunction has been associated with placental pathology and disease states^29–32^, we examined isolectin B4 binding to the placental glycocalyx associated with blood vessels as a proxy for endothelial cells including in decidua to assess isolectin B4 positive cell density and level of expression. Staining tissue sections to detect isolectin B4 binding highlights the extracellular matrix of endothelial cells (among other cell types), allowing visualization of highly vascularized regions within the placenta^33^. At E18.5, both the number of isolectin B4-positive cells and the intensity of staining were blunted (p < 0.05) in ERKdko/ERKdko placentas in both the decidua and junctional zone compared to control (Fig. 5A,B). The ERKdko/control placentas were highly variable with regard to isolectin B4 binding resulting in no statistical differences when compared to the other genotypes (not shown).

### GRIC-mediated recombination of ERK is associated with decreased placental parturition-associated apoptosis

We examined two possible mechanisms that may account for the prolonged gestation/delayed parturition in this model. First, the prolonged gestation interval observed in the ERKdko females was reminiscent of phenotype described for the cyclooxygenase 1 (PGHS1/COX1) knockout animal^34–39^. We speculated that ERK signaling within the decidua and labyrinth may be necessary for the proper expression pattern of COX1 at or near term. As an initial assessment, we used immunohistochemistry to determine if marked differences in COX1 protein expression were detectable in placental sections from control and ERKdko female mice at e18.5 (Supplemental Fig. 3). These studies demonstrated that COX1 immunoreactivity was readily detectable in control and ERKdko pregnancies suggesting that differential expression or deficiencies in COX1 was not likely causal in the context of our model of prolonged gestation.

**Figure 6:**
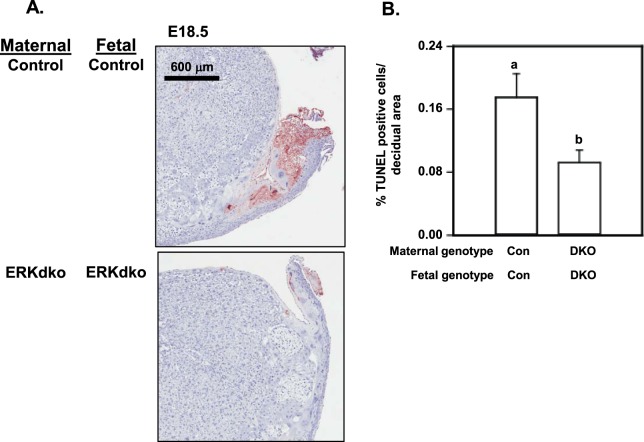
Assessment of TUNEL^+^ cells within the maternal-fetal interface at near term in GRIC ERKdko females. (**A**) Representative images of placentas from control/control and GRIC ERKdko/GRIC ERKdko placentas at e18.5 stained using TUNEL as an indicator of apoptosis (red staining). The magnification bar is shown. (**B**) TUNEL^+^ cells/μm^2^ is reported for control/control and GRIC ERKdko/GRIC ERKdko placentas at e18.5. N = 5 placentas/genotype. Differing letters (a and b) over individual bars identify differences (p < 0.05).

As a second approach, we examined the progression of placental apoptosis within the maternal/fetal interface at the time of onset of parturition (Fig. 6A). Sections from control/control placentas showed focal areas of apoptosis at the interface of the decidua and uterus, putatively allowing for initial separation of the maternal/placental unit at the time of parturition^40–43^. The ERKdko/ERKdko placentas had markedly reduced TUNEL-positive staining around the edges of the decidua at e18.5, indicating a loss or delay of physiologic apoptosis associated with parturition (Fig. 6). Control/control placentas showed positive TUNEL staining in ~17% of the decidual area in this region of the placental disk, while ERKdko/ERKdko placentas showed only ~8% percent of the decidual area was positive for TUNEL staining at e18.5 (p < 0.05, Fig. 6B). TUNEL staining in placentas from ERKdko/control placentas showed an intermediate phenotype and were not significantly different than either of the other two groups (data not shown). The loss of pro-apoptotic tone in ERKdko/ERKdko placentas at E18.5 occurred coincident with prolonged gestation and delayed parturition suggesting that they may be associated.

## Discussion

While loss of ERK signaling within the gonadotrope has been shown to be detrimental to the function of the hypothalamic-pituitary-gonadal axis as well as the placenta, this is the first report of a novel model of placental ERK gene deletion associated with expression of a GnRHR-Cre driver^3,12,13^. In the present studies, the anovulatory phenotype due to loss of pituitary LH production in the GRIC-ERKdko model was overcome through exogenous gonadotropin administration, inducing a subset of ERKdko animals to ovulate and become pregnant. The resulting pregnancy in ERKdko dams was abnormal, characterized by altered placental architecture, prolonged gestation and parturition, and 100% neonatal mortality by postnatal day 3. Based upon the reporter mouse studies, GnRHR-regulated Cre expression and recombination occurred in decidua in midgestation and within the placental labyrinth during later stages of pregnancy. The altered placental architecture in ERKdko/ERKdko placentas occurred coincident with a significant reduction in physiologic apoptosis at e18.5 putatively necessary for placental separation prior to parturition, consistent with the prolonged gestation phenotype observed. Given the complexity of the expression for Cre recombinase in the GRIC model (gonadotropes and placental compartment), we cannot specifically rule out the possibility that loss of gonadotropins contributed to the placental phenotype. Moreover, an important caveat to our studies is that we have not yet demonstrated that conditional ERK deletion specifically within the implantation site (decidua and or labyrinth) is causally or directly related to the abnormal placental architecture or the delayed parturition phenotype. Our understanding of the specific impact of ERK signaling deletion within the implantation site awaits development of new mouse models. Our studies do support the hypothesis that conditional deletion of ERKs within the maternal fetal interface is likely to be associated with the phenotype observed in the present studies. Collectively, these studies shed new light on the potential role of ERK signaling within the reproductive axis including the implantation site in the mouse.

Initial analyses of human endometrium and decidua from early pregnancy revealed the presence of mRNA expression of GnRH but not the GnRHR^44^; however, subsequent studies also examining human tissues reported that endometrial and decidual tissues were responsive the GnRH analogs as measured by induction of phosphoinositol turnover^45^. These observations were consist with findings in the HTR-8/SVneo human extravillous trophoblast cell line where GnRH stimulated expression of several proangiogenic chemokines^46^ and in primary human trophoblasts and decidual stromal cells from term placentas where GnRH stimulated the production of chorionic gonadotropin^23^. Some of the most comprehensive studies of the action of GnRH on placental cell behavior come from Peter Leung’s group. Using primary culture of first trimester trophoblast cells or the extravillous trophoblast model HTR-8/SVneo, this group demonstrated GnRH-induced RUNX2 expression leading to upregulation of RUNX2-dependent matrix metalloproteinases which may modulate the invasive properties of these trophoblast populations^47^. The potential impact of GnRH/GnRHR signaling and the use of GnRHR antagonists on human endometrial stromal cells is controversial since one group suggested that these antagonists did not impact the behavior of the stromal cells^48^; while another group suggested that antagonism of the GnRHR resulted in elevated apoptotic tone in stromal cells^49^.

The ligands GnRH I and II localize to mononucleate villous trophoblasts, and EVTs, but GnRH I is also found in the multinucleated syncytial trophoblast layer in the chorionic villi in humans during the first trimester of pregnancy^50^. Recently, GnRHR has been described in the canine placenta as well. Similar to the human, GnRHR was expressed at higher levels in the uteroplacental tissues compared to areas of the uterus not associated with the zonary placenta (i.e., between implantation sites in this carnivore litter-bearing species). In the canine placenta, GnRHR was found in fetal trophoblasts and maternal decidual cells, and at the surface and glandular epithelial cells in the uterus^25^. These observations are entirely consistent with expression of Cre recombinase in the GRIC model by the endogenous *GnRHR* promoter in the mouse. In the present studies, it is important to note that we do not provide evidence for a direct and specific role for GnRH-directed GnRHR signaling to the ERK pathway within the maternal-fetal interface in mediating the potential effects of ERK deletion. Rather, we argue that this model examines the putative loss of ERK signaling within the maternal-fetal interface leading to a prolonged gestation, where regulation of the ERK signaling pathway within the placenta may be a function of numerous growth peptides and hormones (potentially including GnRH).

Other studies have shown that loss of ERK2 in null animals causes catastrophic defects early in mouse development^10^ or at later developmental stages in the mouse placenta leading to early fetal death^8,11^. In the former studies, specific loss of ERK2 (deletion of exon 2) resulted in embryonic demise at e6.5. Embryonic death occurred coincident with a lack of mesoderm formation and a marked increase in apoptotic tone within the developing embryo. These observations are difficult to reconcile with delayed or absence apoptotic tone in the GRIC ERKdko model at term, but may reflect the timing of ERK loss in the individual models. In the later studies, ERK2 deletion was mediated by disruption of exon 3^8^ or introduction of a neomycin cassette between exons 2 and 3 disrupting splicing of the mature ERK2 mRNA^11^. In both cases, placental competence was compromised resulting in embryonic death later in development (e8.5 to 12.5). All of these studies point to the specific importance of ERK2 during development since in each experimental paradigm, ERK1 did not compensate for the loss of ERK2. Thus it may not be too surprising that loss of ERK signaling within the implantation site in the GRIC model was detrimental to normal pregnancy. However, our studies did not phenocopy the ERK2 null mouse (early fetal demise) suggesting that the timing of ERK2 loss within the placenta and/or the cell types impacted by such loss help to explain the apparent differences in phenotypes comparing the ERK 2 null mouse with the conditional GRIC ERKdko in the present studies.

Loss of placental ERK2 resulted in reduced intensity of isolectin B4 binding within the blood vessel associated glycocalyx, and placental abnormalities that may have contributed to reduced fetal weight in the current studies. We speculate that expression of GnRHR (and Cre) and subsequent loss of ERKs during the formation/expansion of the decidua prior to e12.5 is a likely cause of the changes in placental histology seen at e18.5^51^. Either the change in architecture or loss of ERK2 proteins could impact trophoblast invasion and subsequent maternal spiral artery remodeling, reducing the number of vessels observed in the decidua^52^. Additionally, matrix metalloproteases, which are regulated by GnRH/GnRHR in the placenta, are involved in maternal spiral artery remodeling, leading to another hypothesis for reduced decidual vascularization; however, the specific role of ERK signaling in this situation remains to be elucidated^52,53^. Although speculative, the reduction in the number of isolectin B4-positive cells and the magnitude of isolectin B4 binding may reflect a reduction in endothelial cell populations or functional competence of the microenvironment within the decidua, which may lead to reduced placental transfer between mother and fetus and impact fetal size. Loss of normal placental architecture, general disorganization of the decidua and junctional zone may also contribute to loss of efficient nutrient exchange between the fetus and dam. The direct cause(s) of the alteration in placenta architecture is currently unclear. Interestingly, the most obvious changes are attributable to the finger-like projections of the junctional zone into the labyrinth. Recall that our Rosa26 reporter mice and subsequent immunofluorescence studies revealed that cells within the junctional zone did not undergo apparent genetic recombination. This may support the conclusion that a secreted product of either the maternal decidua or the labyrinth (or both) may be impacting the organization of the cells within the junctional zone. Interestingly, similar junctional zone histological abnormalities have been demonstrated in studies of implantations sites from various forms of assisted reproductive technologies from the Bartolomei group^54^. In these studies, this abnormal placental architecture was associated with disruptions in genomic imprinting. In other studies, genetic disruption of Plac1 (a maternally derived X chromosome-linked gene expressed in trophoblasts) caused similar junctional zone architectural abnormalities^55^. At present, the potential relationship between ERK signaling within the implantation site and aspects of assisted reproduction, genomic imprinting or Plac1 expression is unknown but may provide some important insights into possible mechanisms.

Prolonged gestation and parturition in this model may reflect the local impact of loss of ERKs within the maternal-fetal interface. Multiple factors initiate parturition, including fetal cortisol, maternal drop in progesterone, oxytocin, prostaglandin F2α and other endocrine factors^56–59^. Our initial examination that ERK signaling may have been required for normal expression of COX1 was a logical hypothesis given the prolonged gestation phenotype of the COX1 deficient mouse^34,35,37–39^. However, we did not detect appreciable differences in COX1 protein expression using immunohistochemistry. Alternatively, distinct changes in apoptotic tone at the time of normal parturition were observed suggesting that local loss of ERK signaling may be critical for establishing this aspect of initiation of parturition. Other alternatives include any one of a number of paracrine and/or endocrine signaling events described above. Progesterone withdrawal is a contributing factor to initiation of parturition and the possibility exists that the baseline reduction in progesterone in our model system does not occur in a timely manner or at a level to initiate parturition^60^. The loss of ERK signaling in GnRHR-expressing cells within the maternal-fetal interface may blunt or abrogate the release of PGF2α thus contributing to the delay in onset of parturition^61^. Alteration in oxytocin release is likely not related to the delayed parturition since this peptide is not implicated as an important factor in the onset of parturition in mice^62^. Future studies will examine these possibilities.

This model holds interesting potential as a model of human disease. GnRHR has been shown to be important in placental development and function; it is even expressed at the implantation sites of ectopic pregnancy^63^. Although multiple mouse models have been used to recapitulate idiopathic hypogonadal hypogonadism (IHH), none have focused on loss of function in cells expressing GnRHR and how ERK signaling may be functioning in these tissue compartments^3,64,65^. Up to 40 percent of IHH patients suffer from a GnRHR mutation, which could alter placental function as well^66^. Work by Janet Hall’s lab provides important evidence that although some women with IHH respond appropriately to gonadotropin supplementation and are able to carry a normal pregnancy, others are not^67,68^. Some of these women suffer from multiple miscarriages, most often early in gestation. Not all patients with these mutations showed early pregnancy losses, indicating a non-genetic component, such as alterations in placental function as a potentially contributing factor^67,69,70^. Further investigation of this model system may elucidate missing links and help improve assisted reproduction in women with IHH.

## Materials and Methods

### Animals

Animals were handled in compliance with a protocol approved by the Cornell University Institutional Animal Care and Use Committee. ERK1 null (*ERK1*^−/−^), ERK2 floxed (*ERK2*^*fl/fl*^) and GnRH receptor (GnRHR) IRES Cre (GRIC) mice have been described previously^3,12,13,64^. The presence of the internal ribosome entry site (IRES) allows for co-expression of the GnRHR and Cre recombinase regulated by the endogenous *GnRHR* promoter. For experiments involving analyses of Cre activity, ROSA26-GNZ KI mice were purchased from Jackson Laboratory. This reporter strain was crossed with GRIC animals, and designated ROSA26^GRIC**+**^ or ROSA26^GRIC−^. For timed matings, mice were paired and checked for copulatory plugs daily. Pairs were separated after a copulatory plug was observed (indicating embryonic day (e)0.5). On e10.5, e12.5 and e18.5, the dams were euthanized, and maternal-fetal implantation sites were carefully dissected. For superovulation studies, females were injected with 100 IU of pregnant mare serum gonadotropin (PMSG) intraperitoneally. Approximately 46–48 hours later, they were injected with 100 IU human chorionic gonadotropin (hCG) intraperitoneally. For assessing *corpora lutea* (CL) and follicle formation, superovulated animals were euthanized, and ovaries were collected for histological examination. For induction of pregnancy and assessment of parturition, superovulated control and ERKdko females were placed with fertile males and checked for copulatory plugs the following morning day. Following evidence of copulation, females were minimally handled until e18.5, at which point they were checked every 12 hours for behavioral signs of initiation of parturition, contractions, distress, hiding behaviors, blood on the cage bedding, and pups.

### Genotyping

Genomic DNA was isolated from tail snips, using an E-Z Tissue DNA Kit (Omega Biotek, Norcross, GA) per the manufacturer’s instructions. Routine PCR genotyping was performed on animals as previously described^71^. PCR confirmation of *ERK1* knockout, *ERK2* floxed, *GRIC* and *ROSA26* alleles were performed with primers reported in Table 2.

**Table 2:** PCR primer sets used in genotyping mice.

Primer set		Sequence (5′ to 3′)
Rosa26 Reporter	*Rosa26* Forward	TAA GCC TGC CCA GAA GAC TC
	*Rosa26* Reverse	AAA GTC GCT CTG AGT TGT TAT
	*Rosa26* Common	TCC AGT TCA ACA TCA GCC GCT ACA
ERK1	*Erk1* Forward	AAG GTT AAC ATC CGG TCC AGC A
	*Erk1*Reverse	AAG CAA GGC TAA GCC GTA CC
ERK2	*Erk2* Forward	AGC CAA CAA TCC CAA CCC TG
	*Erk2* Reverse	GGC TGC AAC CAT CTC ACA AT
GnRH Receptor	*Gnrhr* Forward	GAA CTA CAG CTG AAT CAG TC
	*Gnrhr* Reverse	CTC TAA CAA ACT CTG TAC A
	*Gnrhr* Homozygous	CGG AAT TCA TCG ATC ATA TCA GAT CC

### Immunoblotting

Placentas were dissected such that a portion was placed in formalin for histological studies, snap frozen for RNA analysis, or homogenized in lysis buffer containing 20 mM Tris-HCl (pH 8.0), 130 mM NaCl, 10% glycerol, 1% Nonidet P-40, 0.1% sodium dodecyl sulfate, 0.5% deoxycholate, 2 mM EDTA, 5 mM sodium vanadate, 0.2 mM phenylmethysulfonylfluoride, and 5 mM benzamidine. Protein concentrations of lysates were determined by Bradford assay. Samples were boiled for 5 minutes in sodium dodecyl sulfate loading buffer (2x contains 100 mM Tris pH 6.8, 4% sodium dodecyl sulfate, 0.2% bromophenol blue and 200 mM dithiothreitol), resolved by SDS-PAGE, and transferred to polyvinylidine difluoride membranes by electroblotting. Membranes were blocked with 5% nonfat dry milk in TBST (10 mM Tris-HCl, pH 7.5; 150 mM NaCl; 0.05% Tween 20) and then incubated with specified antisera (anti-ERK2, anti-β actin and horseradish peroxidase-conjugated secondary antibodies from Santa Cruz Biotech, Dallas, TX)^3^. Protein bands were visualized using enhanced chemiluminescence according to manufacturer’s instructions (BioRad, Berkeley, CA) and imaged on a ChemiDoc XRS (BioRad, Berkeley, CA) Band densities were analyzed using Image Lab software (BioRad, Berkeley, CA).

### RNA isolation and quantitative PCR

Placentas were subjected to Trizol (ThermoFisher, Waltham, MA) extraction per the manufacturer’s instructions. Reverse transcription of RNA was performed using the High-Capacity cDNA Reverse Transcription Kit (Applied Biosystems, Foster City, CA) according to the manufacturer’s directions. The sequence of the primers used in these amplification studies are reported in Table 3. Amplifications were carried out using a BioRad CFX96 Touch Real-Time OCR Detection System (BioRad, Berkeley, CA). RNA levels were standardized using the internal control Gapdh and calibrated to corresponding transcript levels of a control group by the ΔCt method^3^.

**Table 3:** Quantitative PCR primer sets for assessing expression of GnRH receptor and GAPDH.

Primer set		Sequence (5′ to 3′)
Glyceraldehydes-3-phosphate dehydrogenase	*Gapdh* forward	ATGTTTGTGATGGGTGTGAA
	*Gapdh* reverse	ATGCCAAAGTTGTCATGGAT
GnRH Receptor	*Gnrhr* forward	TGCTCGGCCATCAACAACA
	*Gnrhr* reverse	GGCAGTAGAGAGTAGGAAAAGGA

### Histology, immunofluorescence, Immunohistochemistry and β-galactosidase assays

Mice were paired and checked for copulatory plugs daily. Pairs were separated after a copulatory plug was observed (e0.5). On e12.5 and e18.5, the dams were euthanized, and placentas were carefully dissected. For histological examination, tissues were fixed in 10% neutral-buffered formalin, paraffin embedded, serially sectioned at 4 µm, and stained with hematoxylin and eosin, terminal deoxynucleotidyl transferase dUTP nick end labeling (TUNEL), smooth muscle actin or isolectin B4 using standard techniques as described previously^72^. Sections were scanned and digitized using an Aperio Scanscope (Vista CA). Placental area, areas of decidua, junctional zone and labyrinth layers were quantitated using Aperio software. TUNEL was quantified using the Positive Pixel Algorithm on ImageScope (Leica Biosystems, Buffalo Grove, IL).

For immunofluorescence labeling of GFP, placentas were embedded in Tissue-Tek OCT media (Sakura Finetek, Torrence, CA) and maintained at −80 °C in 2-methylbutane for 24 hours. Sections (10 µM) were cut using a cryotome and stored at −80 °C. Frozen sections were fixed with 4% paraformaldehyde, blocked for one hour, and stained with anti-GFP antiserum (Abcam, Cambridge, United Kingdom) at 1:100 dilution overnight at 4 C followed with a FITC goat-anti-rabbit secondary antibody (Vector Laboratories, Burlingame, CA). Sections were cover slipped with SlowFade Gold with DAPI (Life Technologies, Carlsbad, CA) and imaged on an AxioVision fluorescent microscope with Zen software (Zeiss, Oberkochen, Germany). Sections were also scanned and digitized using an Aperio Scanscope (Vista CA). For immunohistochemistry studies, placental sections were blocked using an antibody-specific HRP/DAB detection kit (per the manufacturer’s instructions; Abcam, Cambridge, United Kingdom) and stained with the PGHS1/COX1 antibody (Abcam, Cambridge, United Kingdom) at 1:50 dilution overnight. Negative controls included sections treated identically except for addition of the primary antibody. Detection of epitopes was carried out using the kit described above. Sections were cover slipped as described above and imaged and digitized using an Aperio Scanscope (Vista, CA).

For β-galactosidase *in vitro* assays, tissues were fixed in 4% paraformaldehyde/PBS for 1 hour at 4 °C, then rinsed 3 times for 30 minutes each in a rinse buffer (100 mM sodium phosphate (pH 7.3), 2 mM MgCl_2_, 0.01% sodium deoxycholate, 0.02% NP-40 (by volume)). Whole tissues or sections were stained overnight in staining buffer (rinse buffer containing 5 mM potassium ferricyanide, 5 mM potassium ferrocyanide, 1 mg/ml X-gal). Tissues or sections were cleared using methyl salicylate, fixed overnight in 10% formalin, then washed with distilled water twice for 30 minutes. They were then dehydrated by sequential ethanol washes (70%, 95%, twice in 100%), then washed in methyl salicylate until the tissue cleared. For liquid β-Galactosidase Enzyme Assay System with Reporter Lysis Buffer (PROMEGA, Madison, WI), tissues were collected and assayed according to the manufacturer’s instructions.

### Statistics

Pairwise comparisons were made by Student’s *t*-test. For comparisons of more than two means, a one way ANOVA was performed and differences were determined by a Duncan’s test. All data are expressed as means ± standard error of the mean. A *p* value of <0.05 was considered statistically significant.

## Supplementary information

Dataset 1

## References

[1] Junttila MR, Li SP, Westermarck J (2008). Phosphatase-mediated crosstalk between MAPK signaling pathways in the regulation of cell survival. FASEB J..

[2] Chang L, Karin M (2001). Mammalian MAP kinase signalling cascades. Nature.

[3] Bliss SP (2009). ERK signaling in the pituitary is required for female but not male fertility. Mol. Endocrinol..

[4] Bliss SP, Navratil AM, Xie J, Roberson MS (2010). GnRH signaling, the gonadotrope and endocrine control of fertility. Front Neuroendocrinol..

[5] Shapiro PS (1998). Activation of the MKK/ERK pathway during somatic cell mitosis: direct interactions of active ERK with kinetochores and regulation of the mitotic 3F3/2 phosphoantigen. J. Cell Biol..

[6] Chambard JC, Lefloch R, Pouyssegur J, Lenormand P (2007). ERK implication in cell cycle regulation. Biochim. Biophys. Acta.

[7] Mazzucchelli C (2002). Knockout of ERK1 MAP kinase enhances synaptic plasticity in the striatum and facilitates striatal-mediated learning and memory. Neuron.

[8] Saba-El-Leil MK (2003). An essential function of the mitogen-activated protein kinase Erk2 in mouse trophoblast development. EMBO Rep..

[9] Pages G (1999). Defective thymocyte maturation in p44 MAP kinase (ERK 1) knockout mice. Science.

[10] Yao Y (2003). Extracellular signal-regulated kinase 2 is necessary for mesoderm differentiation. Proc. Natl. Acad. Sci. USA.

[11] Hatano N (2003). Essential role for ERK2 mitogen-activated protein kinase in placental development. Genes Cells.

[12] Brown JL (2018). Sex- and Age-Specific Impact of ERK Loss Within the Pituitary Gonadotrope in Mice. Endocrinology.

[13] Wen S (2008). Functional characterization of genetically labeled gonadotropes. Endocrinology.

[14] Wierman ME (2012). Extracellular signal-regulated kinase 1 and 2 are not required for GnRH neuron development and normal female reproductive axis function in mice. Neuroendocrinology.

[15] Fan HY (2009). MAPK3/1 (ERK1/2) in ovarian granulosa cells are essential for female fertility. Science.

[16] Beaumont HM, Smith AF (1975). Embryonic mortality during the pre- and post-implantation periods of pregnancy in mature mice after superovulation. J. Reprod. Fertil..

[17] Van DAI, D’Hooghe T (2001). Superovulation of female mice delays embryonic and fetal development. Hum. Reprod..

[18] Fowler RE, Edwards RG (1957). Induction of superovulation and pregnancy in mature mice by gonadotrophins. J. Endocrinol..

[19] Murr SM, Bradford GE, Geschwind II (1974). Plasma luteinizing hormone, follicle-stimulating hormone and prolactin during pregnancy in the mouse. Endocrinology.

[20] Westergaard LG, Laursen SB, Andersen CY (2000). Increased risk of early pregnancy loss by profound suppression of luteinizing hormone during ovarian stimulation in normogonadotrophic women undergoing assisted reproduction. Hum. Reprod..

[21] Labhsetwar AP, Watson DJ (1974). Temporal relationship between secretory patterns of gonadotropins, estrogens, and prostaglandin-F in periparturient rats. Biol. Reprod..

[22] Nir I, Goldhaber G, Hirschmann N, Shani J (1977). Prolactin and luteinizing hormone levels before and after parturition in rats bearing either single or multiple embryos. J. Endocrinol..

[23] Lee HJ (2010). Role of GnRH-GnRH receptor signaling at the maternal-fetal interface. Fertil. Steril..

[24] Kang SK, Tai CJ, Cheng KW, Leung PC (2000). Gonadotropin-releasing hormone activates mitogen-activated protein kinase in human ovarian and placental cells. Mol. Cell Endocrinol..

[25] Schafer-Somi S (2015). GnRH and its receptor (GnRH-R) are expressed in the canine placenta and uterus. Theriogenology.

[26] Chason RJ (2015). GnRH agonist reduces estrogen receptor dimerization in GT1-7 cells: evidence for cross-talk between membrane-initiated estrogen and GnRH signaling. Mol. Cell Endocrinol..

[27] Raga F (1999). The role of gonadotropin-releasing hormone in murine preimplantation embryonic development. Endocrinology.

[28] Raga F (1998). Quantitative gonadotropin-releasing hormone gene expression and immunohistochemical localization in human endometrium throughout the menstrual cycle. Biol. Reprod..

[29] Li M (2013). Fetal-derived adrenomedullin mediates the innate immune milieu of the placenta. J. Clin. Invest.

[30] Adamson SL (2002). Interactions between trophoblast cells and the maternal and fetal circulation in the mouse placenta. Dev. Biol..

[31] Khong TY, De WF, Robertson WB, Brosens I (1986). Inadequate maternal vascular response to placentation in pregnancies complicated by pre-eclampsia and by small-for-gestational age infants. Br. J. Obstet. Gynaecol..

[32] Cross JC (2002). Trophoblast functions, angiogenesis and remodeling of the maternal vasculature in the placenta. Mol. Cell Endocrinol..

[33] Lang I, Hahn T, Dohr G, Skofitsch G, Desoye G (1994). Heterogeneous histochemical reaction pattern of the lectin Bandeiraea (Griffonia) simplicifolia with blood vessels of human full-term placenta. Cell Tissue Res..

[34] Gross GA (1998). Opposing actions of prostaglandins and oxytocin determine the onset of murine labor. Proc. Natl. Acad. Sci. USA.

[35] Loftin CD, Trivedi DB, Langenbach R (2002). Cyclooxygenase-1-selective inhibition prolongs gestation in mice without adverse effects on the ductus arteriosus. J. Clin. Invest.

[36] Langenbach R, Loftin CD, Lee C, Tiano H (1999). Cyclooxygenase-deficient mice. A summary of their characteristics and susceptibilities to inflammation and carcinogenesis. Ann. N. Y. Acad. Sci..

[37] Langenbach R, Loftin C, Lee C, Tiano H (1999). Cyclooxygenase knockout mice: models for elucidating isoform-specific functions. Biochem. Pharmacol..

[38] Burdon C, Mann C, Cindrova-Davies T, Ferguson-Smith AC, Burton GJ (2007). Oxidative stress and the induction of cyclooxygenase enzymes and apoptosis in the murine placenta. Placenta.

[39] Herington JL (2018). Prostaglandin-Endoperoxide Synthase 1 Mediates the Timing of Parturition in Mice Despite Unhindered Uterine Contractility. Endocrinology.

[40] Smith S, Francis R, Guilbert L, Baker PN (2002). Growth factor rescue of cytokine mediated trophoblast apoptosis. Placenta.

[41] Mu J (2003). Apoptosis and related proteins in placenta of intrauterine fetal death in prostaglandin f receptor-deficient mice. Biol. Reprod..

[42] Mu J (2002). Expression of apoptosis in placentae from mice lacking the prostaglandin F receptor. Placenta.

[43] Mu J (2002). Apoptosis and related proteins during parturition in prostaglandin F receptor-deficient mice. Biochem. Biophys. Res. Commun..

[44] Ikeda M, Taga M, Kurogi K, Minaguchi H (1997). Gene expression of gonadotropin-releasing hormone, but not its receptor, in human endometrium and decidua. Mol. Cell Endocrinol..

[45] Takeuchi S, Futamura N, Minoura H, Toyoda N (1998). Possible direct effect of gonadotropin releasing hormone on human endometrium and decidua. Life Sci..

[46] Cavanagh PC (2009). Gonadotropin-releasing hormone-regulated chemokine expression in human placentation. Am. J. Physiol Cell Physiol.

[47] Peng B (2016). GnRH regulates trophoblast invasion via RUNX2-mediated MMP2/9 expression. Mol. Hum. Reprod..

[48] Klemmt PA (2009). Effects of gonadotrophin releasing hormone analogues on human endometrial stromal cells and embryo invasion *in vitro*. Hum. Reprod..

[49] Wu HM (2012). Gonadotrophin-releasing hormone antagonist induces apoptosis in human decidual stromal cells: effect on GADD45alpha and MAPK signaling. Hum. Reprod..

[50] Chou CS, Beristain AG, MacCalman CD, Leung PC (2004). Cellular localization of gonadotropin-releasing hormone (GnRH) I and GnRH II in first-trimester human placenta and decidua. J. Clin. Endocrinol. Metab.

[51] Rossant J, Cross JC (2001). Placental development: lessons from mouse mutants. Nat. Rev. Genet..

[52] Liu J, MacCalman CD, Wang YL, Leung PC (2009). Promotion of human trophoblasts invasion by gonadotropin-releasing hormone (GnRH) I and GnRH II via distinct signaling pathways. Mol. Endocrinol..

[53] Harris LK (2010). Trophoblast- and vascular smooth muscle cell-derived MMP-12 mediates elastolysis during uterine spiral artery remodeling. Am. J. Pathol..

[54] de WE (2015). The cumulative effect of assisted reproduction procedures on placental development and epigenetic perturbations in a mouse model. Hum. Mol. Genet..

[55] Muto M, Fujihara Y, Tobita T, Kiyozumi D, Ikawa M (2016). Lentiviral Vector-Mediated Complementation Restored Fetal Viability but Not Placental Hyperplasia in Plac1-Deficient Mice. Biol. Reprod..

[56] Liggins GC (1968). Premature parturition after infusion of corticotrophin or cortisol into foetal lambs. J. Endocrinol..

[57] Magyar DM (1980). Time-trend analysis of plasma cortisol concentrations in the fetal sheep in relation to parturition. Endocrinology.

[58] Sugimoto Y (1997). Failure of parturition in mice lacking the prostaglandin F receptor. Science.

[59] Kokubu K, Hondo E, Sakaguchi N, Sagara E, Kiso Y (2005). Differentiation and elimination of uterine natural killer cells in delayed implantation and parturition mice. J. Reprod. Dev..

[60] Condon JC, Jeyasuria P, Faust JM, Wilson JW, Mendelson CR (2003). A decline in the levels of progesterone receptor coactivators in the pregnant uterus at term may antagonize progesterone receptor function and contribute to the initiation of parturition. Proc. Natl. Acad. Sci. USA.

[61] Siler-Khodr TM, Khodr GS, Valenzuela G, Harper MJ, Rhode J (1986). GnRH effects on placental hormones during gestation. III. Prostaglandin E, prostaglandin F, and 13,14-dihydro-15-keto-prostaglandin F. Biol. Reprod..

[62] Takayanagi Y (2005). Pervasive social deficits, but normal parturition, in oxytocin receptor-deficient mice. Proc. Natl. Acad. Sci. USA.

[63] Peng B (2016). Gonadotropin-releasing hormone and gonadotropin-releasing hormone receptor are expressed at tubal ectopic pregnancy implantation sites. Fertil. Steril..

[64] Tran S (2013). Impaired fertility and FSH synthesis in gonadotrope-specific Foxl2 knockout mice. Mol. Endocrinol..

[65] Wang H, Hastings R, Miller WL, Kumar TR (2016). Fshβ-iCre mice are efficient and specific Cre deleters for the gonadotrope lineage. Mol. Cell Endocrinol..

[66] Beranova M (2001). Prevalence, phenotypic spectrum, and modes of inheritance of gonadotropin-releasing hormone receptor mutations in idiopathic hypogonadotropic hypogonadism. J. Clin. Endocrinol. Metab.

[67] Seminara SB (2000). Successful use of pulsatile gonadotropin-releasing hormone (GnRH) for ovulation induction and pregnancy in a patient with GnRH receptor mutations. J. Clin. Endocrinol. Metab.

[68] Meysing AU (2004). GNRHR mutations in a woman with idiopathic hypogonadotropic hypogonadism highlight the differential sensitivity of luteinizing hormone and follicle-stimulating hormone to gonadotropin-releasing hormone. J. Clin. Endocrinol. Metab.

[69] Gianetti E (2012). When genetic load does not correlate with phenotypic spectrum: lessons from the GnRH receptor (GNRHR). J. Clin. Endocrinol. Metab.

[70] Zernov N, Skoblov M, Baranova A, Boyarsky K (2016). Mutations in gonadotropin-releasing hormone signaling pathway in two nIHH patients with successful pregnancy outcomes. Reprod. Biol. Endocrinol..

[71] Newbern J (2008). Mouse and human phenotypes indicate a critical conserved role for ERK2 signaling in neural crest development. Proc. Natl. Acad. Sci. USA.

[72] Clark PA (2012). Distal-less 3 haploinsufficiency results in elevated placental oxidative stress and altered fetal growth kinetics in the mouse. Placenta.

