# Supplementary material for: Conditional loss of ERK1 and ERK2 results in abnormal placentation and delayed parturition in the mouse
Source: Sci Rep. 2019 Jul 3;9:9641. doi: 10.1038/s41598-019-45997-0 (PMC6610138; doi:10.1038/s41598-019-45997-0)

Conditional loss of ERK1 and ERK2 results in abnormal placentation and delayed parturition in the mouse

Jessica L. Brown[1], Jennifer L. Sones[1,2], Cynthia N. Angulo[1], Keelin Abbott[1], Andrew D. Miller[1], Ulrich Boehm[3] and Mark S. Roberson[1]

[1]Dept. Biomedical Science, College of Veterinary Medicine, Cornell University, Ithaca, NY
[2]Dept. Veterinary Clinical Sciences, School of Veterinary Medicine, Louisiana State University, Baton Rouge, LA
[3]Experimental Pharmacology, Center for Molecular Signaling (PZMS), Saarland University School of Medicine, Homburg, Germany

Supplementary Figures

Supplemental Figure 1

A.

e12.5

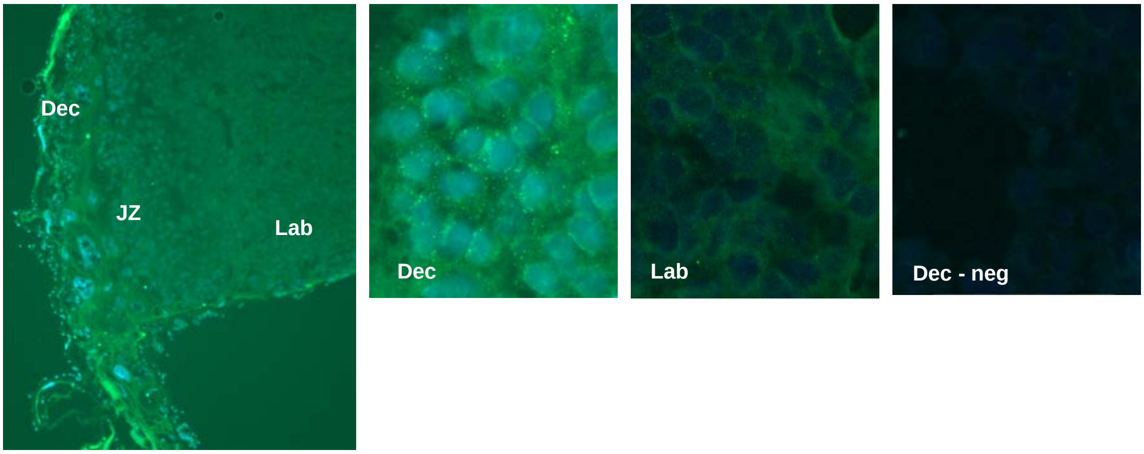

B.

e18.5

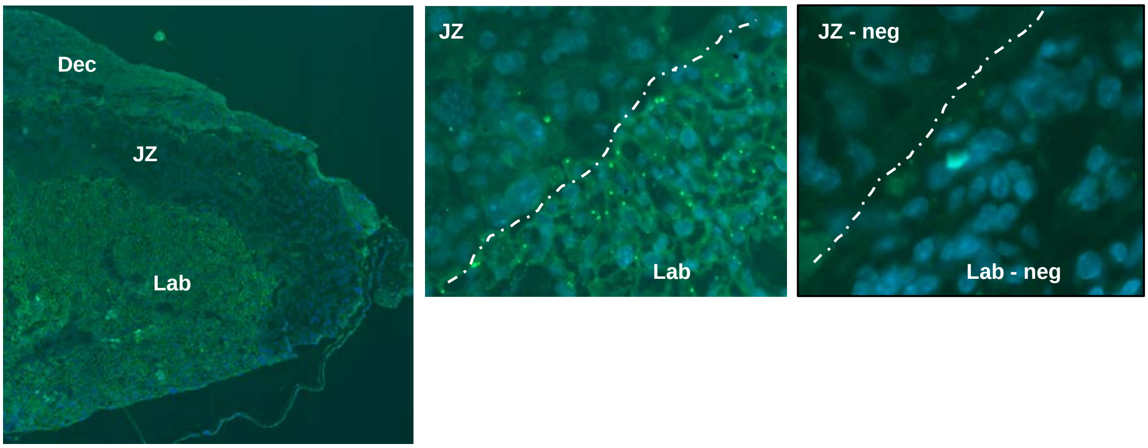

Supplemental Figure 2

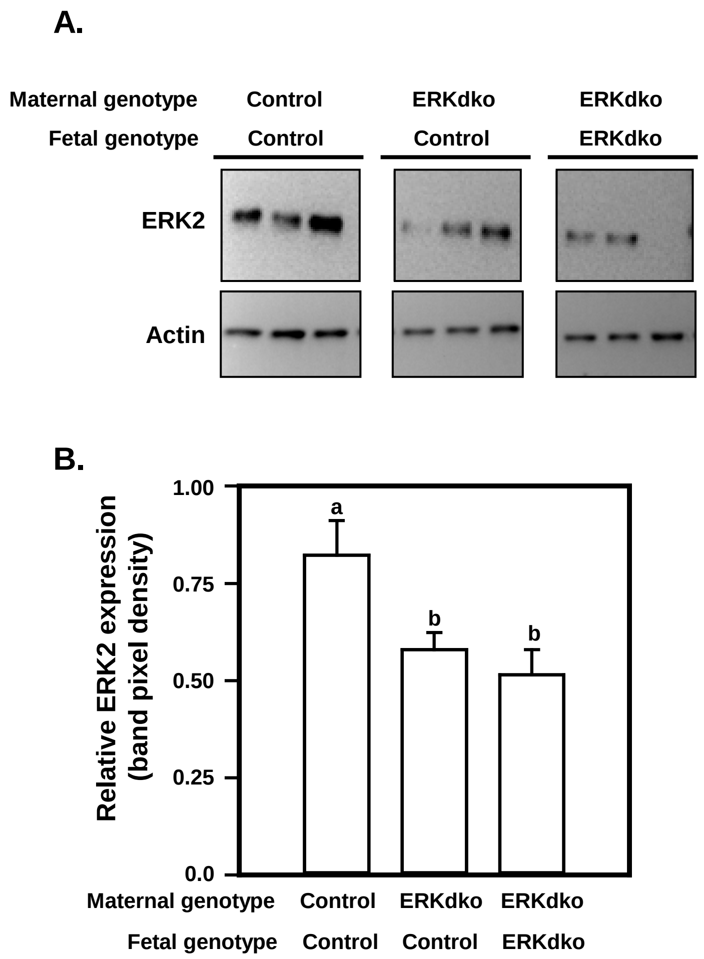

Supplemental Figure 3

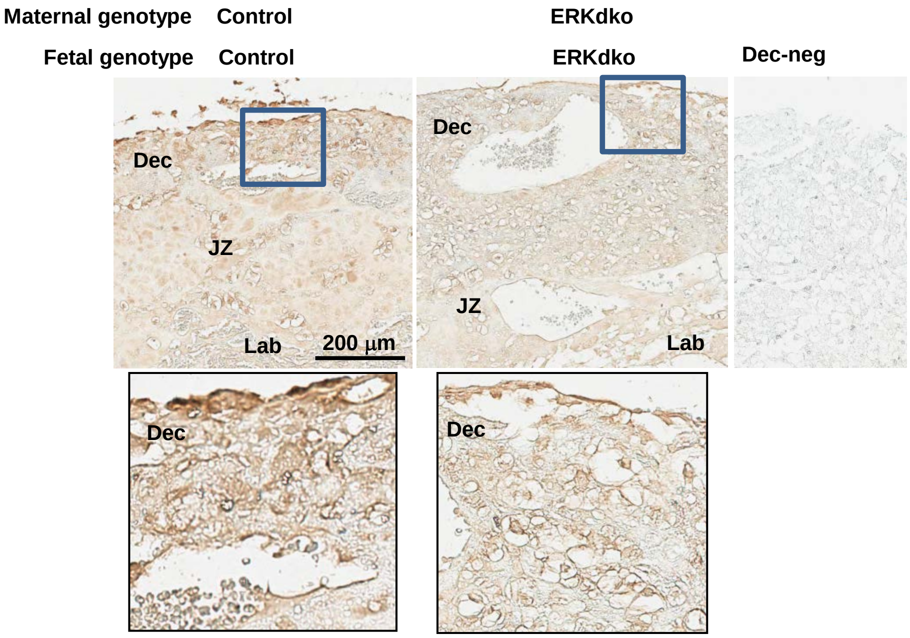

Supplemental Figure 4 - Uncropped western blot gels from Supplementary Figure 2A

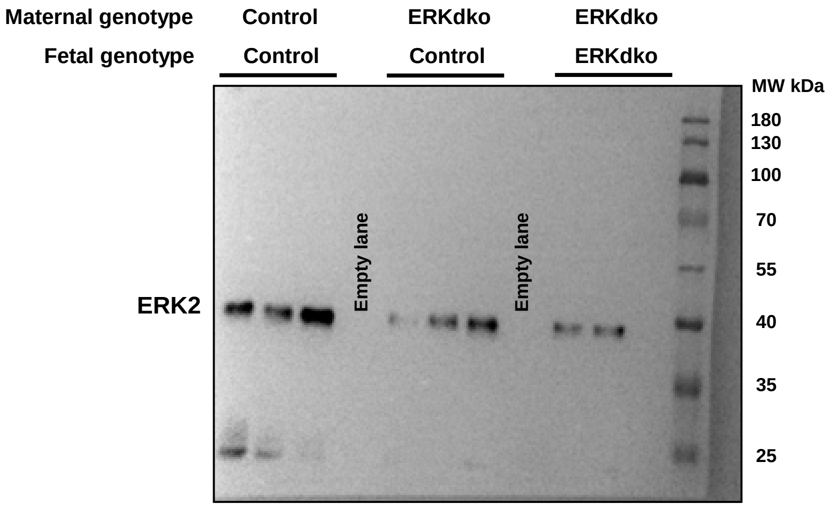

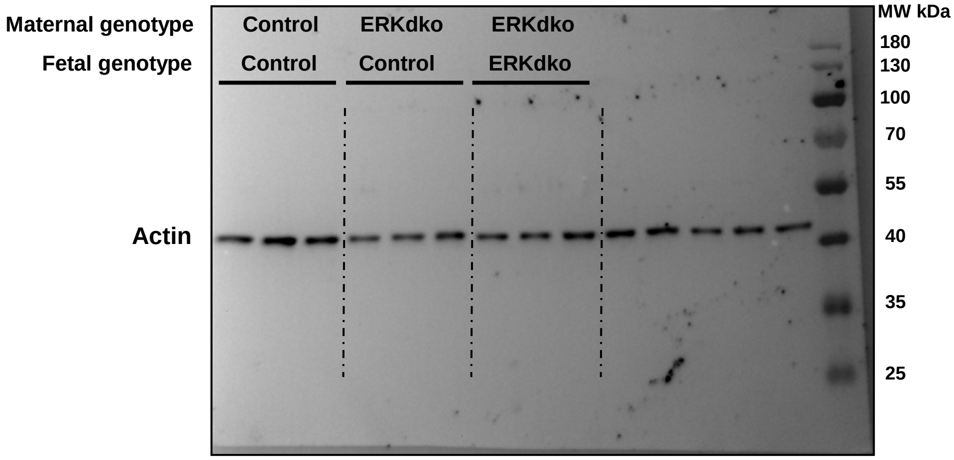

Supplement: Supplementary file 1 — Dataset 1 [file 41598_2019_45997_MOESM1_ESM.pdf]
